# Genetic Diversity, Regional Distribution, and Clinical Characteristics of Severe Fever with Thrombocytopenia Syndrome Virus in Gangwon Province, Korea, a Highly Prevalent Region, 2019–2021

**DOI:** 10.3390/microorganisms11092288

**Published:** 2023-09-11

**Authors:** Mi-Young Moon, Hyeon Kyu Kim, Se-Jin Chung, Jae Hwan Byun, Ha-Na Kim, Woan Lee, Soon-Won Lee, Sezim Monoldorova, Sungkyeong Lee, Bo-Young Jeon, Eun-Joo Lim

**Affiliations:** 1 Infectious Disease Intelligence Division, Gangwon Institute of Health and Environment, Chuncheon 24203, Republic of Korea; moonmy1007@korea.kr (M.-Y.M.);; 2 Department of Biomedical Laboratory Science, College of Digital Healthcare Convergence, Yonsei University, Wonju 26493, Republic of Korea

**Keywords:** severe fever with thrombocytopenia syndrome, severe fever with thrombocytopenia syndrome virus, genotypes, incidence rate

## Abstract

Severe fever with thrombocytopenia syndrome (SFTS) is an arthropod-borne viral disease with a high mortality rate with high fever and thrombocytopenia. We investigated the clinical and epidemiological characteristics and viral genotypes from 2019 to 2021 in Gangwon Province, Korea. Of the 776 suspected cases, 62 were SFTS. The fatality rate was 11.5–28.6% (average rate, 19.4%), and the frequent clinical symptoms were high fever (95.2%), thrombocytopenia (95.2%), and leukopenia (90.3%). Hwacheon had the highest incidence rate per 100,000 persons at 8.03, followed by Inje and Yanggu (7.37 and 5.85, respectively). Goseong, Yangyang, and Hoengseong had rates of 2 or higher; Samcheok, Hongcheon, Jeongsen, and Yeonwol were 1.70–1.98, and Wonju, Gangneung, and Donghae were slightly lower, ranging from 0.31 to 0.74. Of the 57 cases with identified genotypes, eight genotypes (A, B1, B2, B3, C, D, E, and F) were detected, and the B2 genotype accounted for 54.4% (31 cases), followed by the A genotype at 22.8% (13 cases). The B2 and A genotypes were detected throughout Gangwon Province, and other genotypes, B1, B3, C, D, and F, were discovered in a few regions. In particular, genotype A could be further classified into subtypes. In conclusion, SFTS occurred throughout Gangwon Province, and Hwacheon had the highest incidence density. Multiple genotypes of SFTS were identified, with B2 and A being the most common. These findings provide important insights for the understanding and management of SFTS in this region.

## 1. Introduction

Severe fever with thrombocytopenia syndrome (SFTS) is an arthropod-borne viral disease caused by ticks. The Asian long-horned tick, *Haemaphysalis longicornis*, is the main vector of SFTS. The main symptoms of SFTS are high fever, thrombocytopenia, leukopenia, and gastrointestinal symptoms such as nausea, vomiting, and diarrhea. In severe cases, multiple organ failure can lead to death, resulting in high mortality [1].

Dabie bandavirus (previously severe fever with thrombocytopenia syndrome virus, SFTSV) is a negative-stranded RNA virus belonging to the family Phenuiviridae (formerly Bunyaviridae), which consists of three segments: large (L), medium (M), and small (S). SFTSV invades the lymph nodes adjacent to the tick bite site, inhibits B cells to prevent antibody production [2], and causes the virus to proliferate, inducing systemic inflammatory response syndrome [3,4,5]. In addition, thrombocytopenia, one of the main symptoms, is caused by the removal of SFTS-bound platelets in splenic macrophages and the rapid depletion of platelets due to SFTSV-induced coagulation of the vascular endothelium [6,7].

SFTS was first reported in China in 2011 [8] and frequently occurs in East Asia, including Korea, China, and Japan; it has been reported in Vietnam and Taiwan [9,10,11]. In Korea, SFTS was first reported in 2013 in a patient who died of multiple organ failure in Chuncheon, Gangwon Province [12].

SFTSV genotypes were classified into six genotypes (A to F), and genotype B was further divided into three genotypes (B-1, B-2, and B-3) [13,14]. In Korea, genotypes A, B1, B2, B3, D, and F have been reported, with the main genotype being genotype B1 [14,15,16]. Similar to Korea, the main SFTS genotype in Japan is B [13,17]. Genotypes A, D, and F are mainly distributed in mainland China, and genotype B has only been reported in Zhoushan, Eastern China [13,17]. In addition, SFTSV has diverse genotypes due to gene rearrangements [14,17]. The genetic diversity of SFTSV raises concerns regarding antigenic and pathogenic alterations in the virus and requires constant monitoring. Although the overall genotypes of SFTS isolates have been reported [14], there is little data on the distribution of SFTS genotypes in regions where they occur frequently.

Since its first report in Gangwon Province in 2013, SFTS has occurred nationwide, and 1696 cases of SFTS have been reported up to 2022 [18]. Of these, Gangwon Province had 230 cases (13.5%), which was the third highest after Gyeonggi Province (285 cases, 16.8%) and Gyeongbuk Province (242 cases, 14.2%). In addition, the incidence rate per 100,000 persons in Gangwon Province was 1.49, the second highest after Jeju Island.

In this study, the SFTSV genotypes of patients with SFTS in Gangwon Province were analyzed by region, and the correlation between these genotypes, clinical characteristics, and mortality of patients with SFTS was investigated.

## 2. Materials and Methods

### 2.1. Specimen Collection from Patients with Suspected SFTS and Subject Characterization

This study was approved by the Institutional Review Board (IRB) of Yonsei University Mirae Campus (IRB No. 1041849-202208-BR-147-01), which waived the requirement for informed consent. A total of 776 serum specimens were collected from clinically suspected patients with SFTS requested by hospitals or medical institutions in Gangwon Province between 2019 and 2021. The samples were stored at −80 °C until use. The selection criteria for this study were as follows: (1) patients whose administrative address was in Gangwon Province; (2) patients with epidemiological reports; and (3) patients diagnosed with SFTS who had clinical symptoms and met the criteria of the diagnostic guidelines of the Institution of Health and Environment of Gangwon Province as follow. The SFTS infection was confirmed by the detection of M segment and S segment genes of SFTSV using one-step real-time reverse transcription PCR (real-time RT-PCR) (PowerChek SFTSV; KOGENEBIOTECH, Seoul, Republic of Korea). In brief, the partial sequences of M segment and S segment of SFTSV were amplified under the following conditions: 30 min at 50 °C, 10 min at 95 °C, 45 cycles of 15 s at 95 °C, and 30 s at 60 °C. A cycle threshold (Ct) value of 35 or less was designated as positive.

### 2.2. Viral RNA Extraction, Real-Time PCR, and Sequencing

Viral RNA was extracted from the sera using the QIAamp Viral RNA Mini Kit (Qiagen, Inc., Hilden, Germany) according to the manufacturer’s protocol. SFTSV infection was confirmed using a one-step reverse transcription PCR (RT-PCR) with Maxime™ RT-PCR premix (iNtRON Biotechnology Inc., Seongnam, Republic of Korea). In brief, the partial M segment of SFTSV was amplified under the following conditions according to the manufacturer’s instructions: an initial step of 10 min at 50 °C, 3 min at 95 °C for enzyme activation, 30 cycles of 20 s at 95 °C, 20 s at 60 °C, and 40 s at 72 °C, and a final extension step of 5 min at 72 °C. The primer set for the M segment was MF3 [5′-GATGAGATGGTCCATGCTGATTCT-3′] and MR2 [5′-CTCATGGGGTGGAATGTCCTCAC-3′] [13]. PCR products were analyzed using electrophoresis on a 1.5% agarose gel and sequenced with the ABI Prism BigDye (ThermoFisher Scientific Inc., Waltham, MA, UAS) by Macrogen (Macrogen Inc., Seoul, Republic of Korea). The sequences were assembled using the SeqMan Software (DNASTAR, Lasergene version 7, Madison, WI, USA). All SFTSV partial sequences were submitted to GeneBank and assigned the accession numbers.

### 2.3. Phylogenetic Analysis

The phylogenetic analysis was performed using the maximum likelihood tree method [19] and bootstrap [20] based on the Tamura-Nei model [21] using MEGA X (version 7.0). Bootstrap and maximum composite likelihood were performed for phylogenetic testing and substitution models with 1000 permutations, respectively. 

### 2.4. Statistical Analysis

Within each group, frequency (percentage) was summarized for categorical variables, and median and interquartile range (IQR) were summarized by nonparametric quantitative variables. An intergroup comparison was made by Fisher’s exact test or nonparametric Mann-Whitney *U* test. Statistical analyses were performed using GraphPad Prism 7.0 (GraphPad Software, San Diego, CA, USA). A *p*-value less than 0.05 was considered statistically significant.

## 3. Results

### 3.1. General Epidemiological Characteristics of SFTS Cases in Gangwon Province

Between 2019 and 2021, 776 serum specimens were collected from patients with suspected SFTS who were admitted to hospitals in Gangwon Province, Korea. Of these specimens, 73 were confirmed to be SFTSV-positive using RT-PCR. However, nine cases from other provinces and two without epidemiological information were excluded.

**Figure 1:**
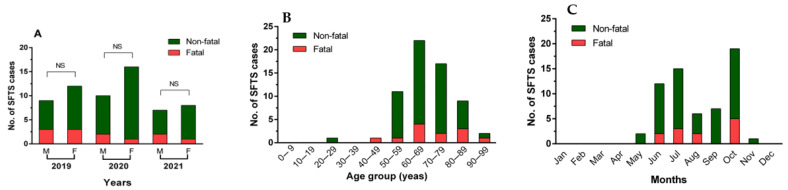
Distribution of severe fever with thrombocytopenia syndrome (SFTS) cases in Gangwon Province from 2019 to 2021. (**A**) Gender, (**B**) age, and (**C**) monthly distribution of the confirmed SFTS cases. M, male; F, female; NS, not significant. The statistically significant differences in fatality rates between each gender and age group of SFTS cases were determined by the Mann-Whitney *U* test.

A total of 62 patients with SFTS were diagnosed between 2019 and 2022 in Gangwon Province, with 21 cases in 2019, 26 in 2020, and 15 in 2022 (Figure 1A). The incidence of SFTS per 100,000 persons was 1.34. The case fatality rates were 28.6% (6 of 21) in 2019, 11.5% (3 of 26) in 2020, and 20.0% (3 of 15) in 2021, with an overall fatality rate of 19.4% (12 of 62).

The clinical characteristics of patients with SFTS were high fever (95.2%, 59 of 62), thrombocytopenia (95.2%, 59 of 62), leukopenia (90.3%, 56 of 62), fatigue (50.5%, 31 of 62), and myalgia (43.5%, 27 of 62) (Table 1). Mental disorders were observed in 21.0% (13 of 62) of SFTS cases, and their incidence was significantly higher in fatal cases (*p* < 0.01).

Of the 62 patients with SFTS, 26 (41.9%) were male, and 36 (58.1%) were female. The incidence of SFTS was slightly higher in females. However, this difference was not statistically significant. The fatality rates in males and females were 26.9% (7 of 26) and 13.9% (5 of 36), respectively. Although males appeared to have a higher fatality rate than females, there was no significant sex difference (Table 1 and Figure 1A).

The patients’ age with SFTS ranged from 25 to 94 years, with a median age of 67. Most patients with SFTS were over 50 years old (96.8%), with the highest frequency of 60–69 years of age (35.5%, 22 of 62), followed by 70–79 years of age (27.4%, 17 of 62) and 50–59 years of age (17.7%, 11 of 62). Most fatal cases were observed in patients aged >60 years (83.3%, 10 of 12), with the highest frequency in those aged 70–79 years (50.0%, 6 of 12). No significant difference was observed in fatality rates between those over 60 years of age (20.0%, 10 of 50) and those under 60 years of age (16.7%, 2 of 12) (Figure 1).

The seasonal distribution of SFTS cases is shown in Figure 1C. Most of the cases occurred between June and October. In particular, 12 cases (19.4%) occurred in June, 15 (24.2%) in July, and 19 (30.6%) in October, accounting for 74.2% of all outbreaks. However, there was a slight decrease to six cases (9.7%) and seven cases (11.3%) in August and September, respectively, which seems to be related to the rainy season. The overall fatality rate was 19.4% (12 of 62); by month, it was 16.7% (2 of 12) in June, 20.0% (3 of 15) in July, 33.3% (2 of 6) in August, and 26.3% (5 of 19) in October, with no significant differences. No deaths were observed among patients with SFTS in September.

### 3.2. Geographical Features of SFTS Outbreaks

SFTS occurred in most areas of Gangwon Province, with Chuncheon having 13 cases (21.0%), followed by Inje with 7 cases (11.3%) and Hwacheon with 6 cases (9.7%) (Table 2). SFTS is observed in most areas of Gangwon-do. No cases of SFTS were observed in Taebaek, Sokcho, or Cherwon.

**Figure 2:**
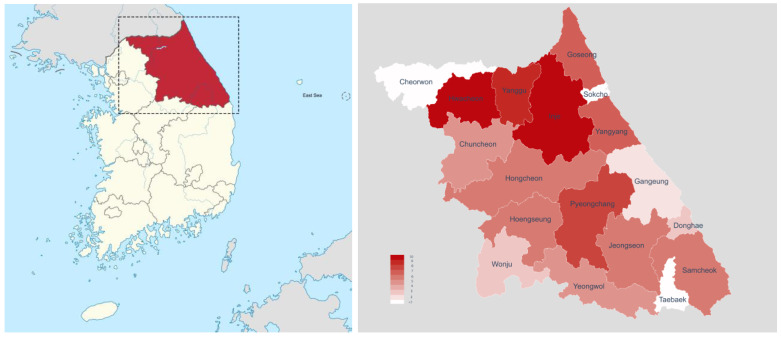
SFTS incidences by population density in Gangwon Province from 2019 to 2021. The incidence rate of SFTS by population was calculated as the number of SFTS cases per 100,000 persons. The map was created using the Statistical Geographic Information Service (SGIS) of Statistics Korea (https://sgis.kostat.go.kr/jsp/english/thematic.jsp, accessed on 1 July 2023).

In terms of SFTS incidence by population density, Hwacheon had the highest incidence rate of 8.03 cases per 100,000 persons, followed by Inje with 7.37 cases per 100,000 persons, Yanggu with 5.86 cases per 100,000 persons, and Pyeongchang with 3.96 cases per 100,000 persons (Figure 2). The incidence rates in Goseong, Yangyang, and Hoengseong ranged from 2.15 to 2.45 per 100,000 persons. In Samcheok, Hongcheon, Jeongseon, and Youngwol, the incidence rate was 1.70–1.98 cases per 100,000 persons. In contrast, among urban areas, Chuncheon had a slightly higher rate of 1.42 cases per 100,000 persons, while Wonju, Gangneung, and Donghae had 0.31–0.74 cases per 100,000 persons.

Regarding the regional fatality rate of SFTS cases, Pyeongchang had the highest fatality rate of 60% (3 out of 5 deaths), followed by 50% (1 out of 2) and 40% (2 out of 5) in Yeongwol and Wonju, respectively. Chuncheon, which had the highest number of SFTS cases, had a fatality rate of 33.3% (4 out of 12). No deaths occurred in the other areas. However, there was no statistically significant difference in the fatality rate by region.

### 3.3. Genotypes of SFTSVs

**Figure 3:**
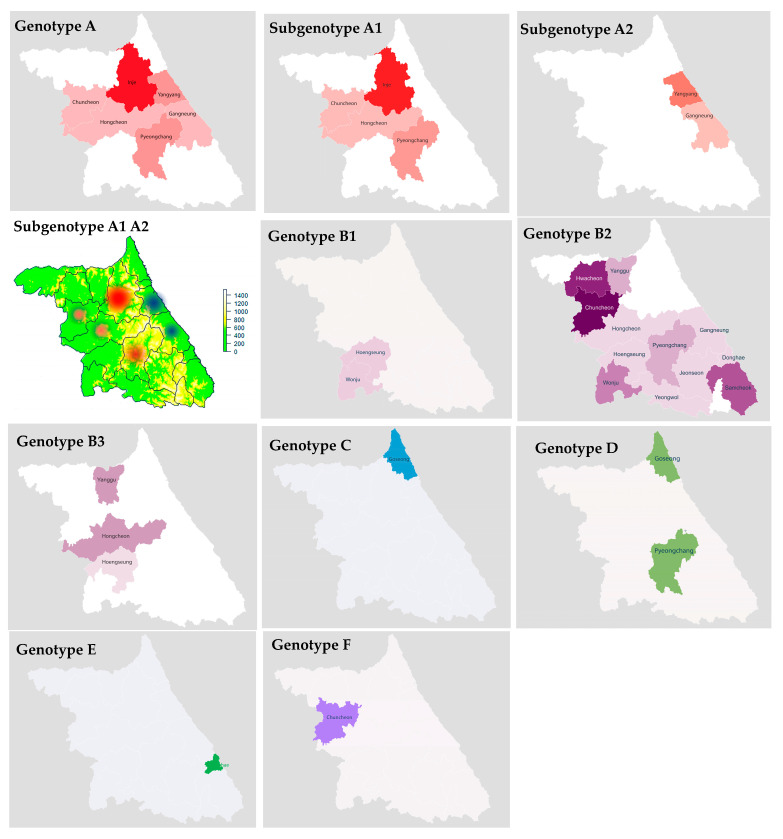
Geographical distribution of SFTSV genotypes in Gangwon Province from 2019 to 2021. The distribution of SFTSV genotypes is shown by region. In the case of genotype A, distribution of subgenotypes A1 and A2 according to the phylogenetic relationship is shown, and their distribution (red circle, A1; blue circle, A2) is also shown geographically.

The SFTSV genotypes were analyzed for SFTS cases from 2019 to 2021. The M-segment sequence was amplified in 57 of the 62 SFTS cases and classified into eight genotypes: A, B1, B2, B3, C, E, D, and F (Table 2 and Table 3). Of the 57 SFTSV cases with identified SFTSV genotypes, genotype B2 was the most frequent, with 31 cases, accounting for 54.4%, followed by genotype A (13 cases, 22.8%) and genotype B3 (5 cases, 8.8%). Genotypes B1, D, and F accounted for 3.2% with 2 cases, and genotypes C and E were identified in one case each. Multiple genotypes were identified in each region (Table 2 and Figure 3). Four genotypes (A, B2, B3, and F) were identified in Chuncheon. Three genotypes coexisted in Hongcheon, Hoengseong, and Pyeongchang; two were detected in Wonju, Gangneung, Donghae, Yanggu, and Goseong. In contrast, only one genotype was detected in Hwacheon, Inje, Samcheok, Yangyang, Yeongwol, and Jeongseon.

Genotypes B2 and A were distributed throughout Gangwon Province (Table 2 and Figure 3). Genotype B1 was distributed in Wonju and Heongseong, the southwestern regions of Gangwon Province. In contrast, genotype B3 was distributed in Hoengseong, Honcheon, Chuncheon, and Yanggu, the western regions of the Taebaek Mountains. Genotype D was detected in Pyeongchang and Goseng. Genotypes C, E, and F were detected only in Goseong, Donghae, and Chuncheon, respectively.

The fatality rate by genotype ranged from 0 to 50%, and the fatality rates of the major genotypes A and B2 were 23.1% (3 of 13) and 19.4% (6 of 31), respectively (Table 3). No significant differences were observed in fatality rates among the genotypes.

**Table 2:** Genetic distribution of severe fever with thrombocytopenia syndrome virus in Gangwon Province from 2019 to 2021.

Region			Genotype							Sum
	A	B1	B2	B3	C	D	E	F	ND	
Chuncheon	1		8					2	1	13
Wonju		1	3						1	5
Gangneung	1		1							2
Donghae			1				1			2
Samcheok			4							4
Hongcheon	1		1	2						4
Heongseung		1	1	1						3
Yeongwol			1						1	2
Pyeongchang	2		2			1				5
Jeongseon			1						1	2
Hwacheon			6							6
Yanggu			2	2						4
Inje	6								1	7
Goseong					1	1				2
Yangyang	2									2
Total	13	2	31	5	1	2	1	2	5	62

**Table 3:** Genotypes of severe fever with thrombocytopenia syndrome virus in Gangwon Province from 2019 to 2021.

Genotype	Nonfatal	Fatal	Total (%)
A	10	3	13 (21.0)
B1	1	1	2 (3.2)
B2	25	6	31 (50.0)
B3	5	0	5 (8.1)
C	1	0	1 (1.6)
D	1	1	2 (3.2)
E	1	0	1 (1.6)
F	2	0	2 (3.2)
Not determined	4	1	5 (8.1)
Sum	50	12	62 (100.0)

### 3.4. Phylogenetic Analysis of SFTSVs

**Figure 4:**
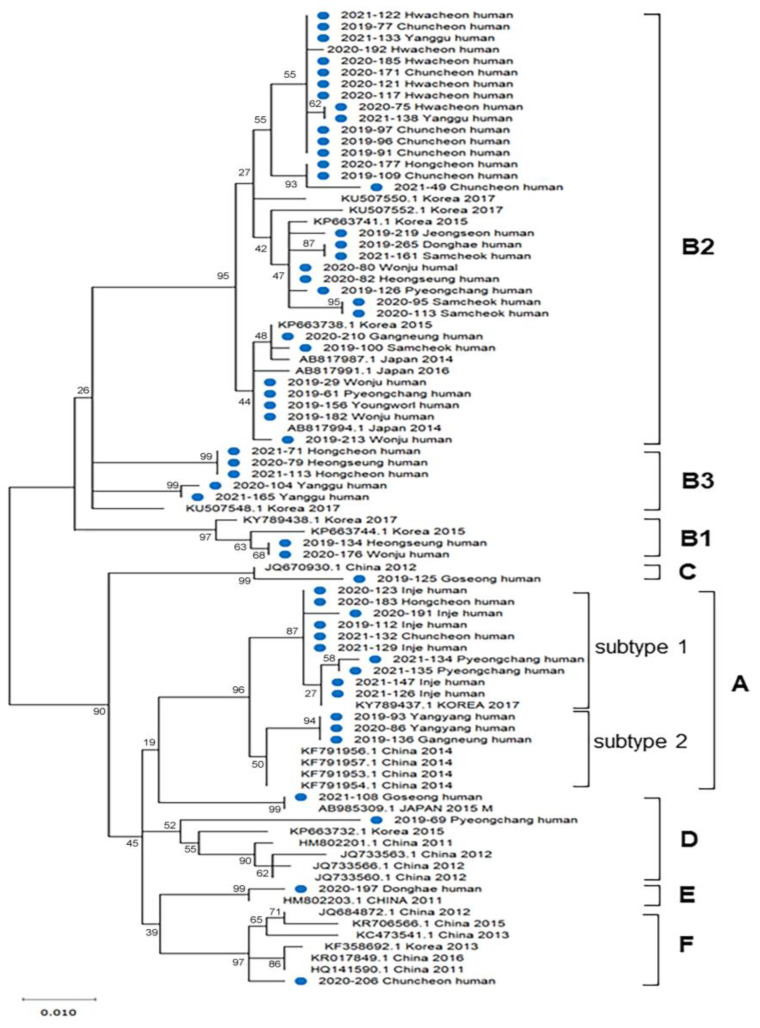
Phylogenetic analysis of SFTSV in Gangwon Province from 2019 to 2021. Phylogenetic analysis based on the partial M segment of SFTSV strains using the maximum likelihood (ML) method based on the Tamura–Nei model. The SFTSV genotypes were assigned to A–F according to the previous studies [13,14,16,17]. The scale bar indicates the nucleotide substitutions per site. The human-derived SFTSV strains analyzed in this study are marked with a blue circle.

A phylogenetic tree was constructed using maximum likelihood to compare the phylogenetic relationships of the SFTSVs. A total of 57 partial sequences of the SFTS M segment, including eight M-segment sequences obtained from patients with SFTS in this study and 28 M-segment sequences from NCBI GenBank, were used for phylogenetic analysis (Figure 4). The genotype B2 sequences detected in Gangwon Province were close to the SFTSV sequence of Korean patients (KY507550.1, KP663741.1, KP663738.1). Interestingly, B2 sequences detected in Hwacheon (2020-75, OR392486; 2020-185, OR392501; 2020-192, OR392503; 2021-122, OR392512), Yanggu (2021-133, OR392516; 2021-138, OR392519), Hongcheon (2020-177, OR392499), and Chuncheon (2019-77, OR392469; 2019-91, OR392470; 2019-96, OR392472; 2019-97, OR392473; 2019-109, OR392475; 2020-171, OR392497; 2021-49, OR392508) formed a new cluster.

The genotype B3 sequence (2021-71, OR392509; 2020-79, OR392487; 2021-113, OR392511; 2020-104, OR392492; 2021-165, OR392522) was close to KU507548.1 (Korea), and B1 sequences (2019-134, OR392479; 2020-176, OR392498) were KP663744.1 (Korea).

Interestingly, genotype A was divided into two clusters (Figure 4). One was the genotype A sequences detected in Inje, Chuncheon, Hongcheon and Pyeongchang, and the other in Gangneung and Yangyang, respectively, name subgenotype 1 and 2. The subgenotype A1 sequences detected in Inje (2019-112, OR392476; 2020-191, OR392502; 2020-123, OR392496; 2021-126, OR392513; 2021-129, OR392514; 2021-147, OR392520), Hongcheon (2020-183, OR392500), Chuncheon (2021-132, OR392515), and Pyeongchang (2021-135, OR392518) were close to KY789437.1, which was reported in Korean patients with SFTS. And the other subtype A2 sequences in Yangyang (2019-93, OR392471; 2020-86, OR392490) and Gangneung (2019-136, OR392480) were close to the genotype A sequences (KF791956.1, KF791957.1, KF791953.1, KF791954.1) reported in China. In particular, they were not only distinguished genetically but also geographically divided by the TaeBaek Mountains (Figure 3).

Genotype C sequence (2019-125, OR392477) detected in Goseong was close to JQ670930.1 (China). Genotype D sequence (Goseong, 2021-108, OR392510) was close to AB985309.1 (Japan). On the other hand, the sequence (2019-69, OR392468) detected in Pyeongchang was somewhat close to KP663732.1 (Korea) and HM802201.1 (China). The genotype E sequence (Donghae, 2020-197, OR392504) was close to HM802203.1 (China). And the F sequence detected in Chuncheon was close to KF358692.1 (Korea) and Chinese sequences such as HM802203.1.

## 4. Discussion

In this study, we investigated the clinical and epidemiological characteristics of SFTS cases in Gangwon Province, a highly endemic area, between 2019 and 2021 and examined the genetic diversity and the regional distribution of the SFTSV genotypes.

The fatality rate of SFTS cases was 19.4% (12 of 62 cases) and was higher in males (26.9%) than females (13.9%); however, the difference was not significant. Most patients were over 50 years of age (98.4%). In the first report of SFTS cases in China, patients aged 50 years and older accounted for 75%, and the fatality rate was 12% [8]. In Korea, the fatality rate of SFTS cases between 2013 and 2015 was 32%, with 85.3% of cases aged 50 years or older [22], and in the Yun et al. study of SFTS cases between 2013 and 2017, the mortality rate was 21%, with no difference between male and females [14]. The findings of this study were similar to those of previous studies [8,12,14,22,23,24], and the variability in fatality rate may be due to increased awareness of SFTS leading to accurate diagnosis and early treatment.

SFTS occurred throughout Gangwon Province, with high incidence in the populous cities of Chuncheon (12 of 62 cases, 12.9%) and Wonju (5 cases, 9.7%), but also in mountainous regions such as Inje (7 cases, 11.3%), Hwacheon (6 cases, 9.7%), and Pyeongchang (5 cases, 8.1%). In particular, the highest incidence rate per 100,000 persons was in Hwacheon (8.03), which was approximately five times higher than that in Jeju Province (1.65 per 100,000 persons). This may be the highest known incidence rate in Korea. In addition, Inje and Yanggu showed high incidence rates of 7.37 and 5.85 per 100,000 persons, respectively. In many regions, incidence rates were over 1.7 per 100,000 persons, and only a few regions had slightly lower rates of 0.31–0.74. These results suggest that most of the regions of Gangwon Province are considered SFTS high-risk areas.

When the SFTSV genotype was analyzed in SFTS cases in Gangwon Province, all eight known genotypes were detected in the Province. The B2 genotype accounted for more than half (54.3%, 31 of 57), followed by genotype A (22.8%, 13 of 57). Genotypes B2 and A accounted for 75.4% of the total SFTSV and were the main genotypes in Gangwon Province. These two main genotypes were also distributed throughout Gangwon Province. Genotype B3 was 8.8% (6 of 57) and was detected in the western regions of Gangwon Province. Genotypes B1, C, D, E, and F were detected only in some regions. In a study by Yun et al., genotype B2 was the most prevalent (36.1%) in patients with SFTS nationwide, followed by genotypes B3 (21.1%) and B1 (12.0%). Genotype A accounted for 7.5%, and genotypes F and D accounted for 6.8% and 3.8%, respectively [10]. Considering that Yun et al.’s study analyzed patients with SFTS nationwide, their findings align with ours, where genotype B2 was the dominant genotype in Korea and had a higher rate of more than 50% in Gangwon Province. Moreover, the proportion of genotype B3 was low; however, genotype A accounted for a higher proportion (22.4%). In particular, all samples belonged to genotype A in Inje.

In the phylogenetic analysis, interestingly, genotype A was divided into two subgenotypes, A1 and A2, in the phylogenetic analysis (Figure 3), and these subtypes were also separated geographically. Subgenotype A1 sequences from Inje, Chuncheon, and Pyeongchang were close to previously reported genotype A in Korea. On the other hand, A2 sequences from Gangneung and Yangyang were close to genotype A sequences of China. This implies that the genetic diversity of SFTSV might be associated with natural environments. In addition, genotype B2 sequences in Gangwon Province were close to previously reported SFTSV sequences (KY507550.1, KP663741.1, KP663738.1) of Korean patients, and some B2 sequences detected in Hwacheon, Yanggu, Hongcheon, and Chuncheon formed a new cluster. It might be necessary to be elucidated with further extensive studies.

The occurrence of SFTS can be related to various factors, including the human host, the environment, and the vector. *H*. *longicornis* is distributed throughout East Asia, including Korea, China, and Japan; however, it has recently been reported in the United States [25,26,27] and is known as the main transmitter vector of SFTS. The tick is a blood-sucking arthropod that attaches to and sucks blood from wild animals, such as water deer, wild boar, raccoon dogs, and wild rodents. The natural environment serves as an important habitat for wild animals, and its close association with ticks, the main vector of SFTS, directly impacts the occurrence of SFTS [22]. Gangwon Province is home to a forest area of 1.3 million hectares, accounting for approximately 82% of the total area of the province. In addition, Hwacheon and Inje are close to demilitarized zones where nature is well preserved, thus providing hospitable environments for wild animals. Therefore, natural environments are closely related to the high incidence of SFTS in these areas. Further studies are required to investigate the regional association between tick-derived SFTSV and SFTSV isolates from patients.

## 5. Conclusions

In Gangwon Province, there were 62 SFTS cases from 2019 to 2021, with an average fatality rate of 19.4% (12 of 62), mainly among people over 60 years of age. SFTS occurred throughout Gangwon Province, with Chuncheon having the highest number of cases at 13 cases (21.0%). The incidence rates per 100,000 persons were high throughout Gangwon Province, with Hwacheon having the highest incidence rate of 8.03 cases per 100,000 persons, followed by Inje and Yanggu, 7.37 and 5.86 cases per 100,000 persons, respectively. In the genotype analysis, eight SFTSV genotypes (A, B1, B2, B3, C, D, E, and F), were detected, and the B2 and A genotypes, the predominant genotypes, accounted for 54.4% (31 of 57) and 22.4% (13 of 57), respectively. In the phylogenetic analysis, A genotypes were divided into two genetically and geographically distinct subgenotypes. The incidences of SFTS were high throughout Gangwon Province, suggesting a high risk of SFTS. There was diverse SFTSV genotypes in Gangwon Province, and the predominant genotypes, B2 and A, were widely distributed, while miner genotypes were scattered regionally. 

## Figures and Tables

**Table 1:** Baseline characteristics of patients with severe fever with thrombocytopenia syndrome in Gangwon Province, Korea, from 2019 to 2021.

Characteristics		Non-Fatal (*n* = 50)	Fatal (*n* = 12)	Total (*n* = 62)	*p*-Value
Sex *	Male	19 (38.0)	7 (58.3)	26 (41.9)	0.329
	Female	31 (62.0)	5 (41.7)	36 (58.1)	
Age, years (median; IQR) ^†^		66 (60–77)	72.5 (61–82)	67 (60–78)	0.313
General	Fever	47 (94.0)	12 (100)	59 (95.2)	0.904
Symptoms *	Fatigue	25 (50.0)	6 (50.0)	31 (50.0)	1.000
	Myalgia	22 (44.0)	5 (41.7)	27 (43.5)	1.000
	Headache	13 (26.0)	3 (25.0)	16 (25.8)	1.000
	Arthralgia	9 (18.0)	1 (8.3)	10 (16.1)	0.670
Gastrointestinal	Nausea	9 (18.0)	4 (33.3)	13 (21.0)	0.256
Symptoms *	Vomiting	6 (12.0)	4 (33.3)	10 (16.1)	0.091
	Diarrhea	17 (34.0)	2 (16.7)	19 (30.6)	0.313
	Abdominal pain	15 (30.0)	4 (33.3)	19 (30.6)	1.000
	Anorexia	17 (34.0)	2 (16.7)	19 (30.6)	0.313
Respiratory	Cough	6 (12.0)	1 (8.3)	7 (11.3)	1.000
Symptoms *	Sputum	2 (4.0)	2 (16.7)	4 (6.5)	0.166
	Others	5 (10.0)	1 (8.3)	(6 (9.7)	1.000
Nervous	Mental deterioration	6 (12.0)	7 (58.3)	13 (21.0)	0.002
Symptoms *	Others	4 (8.0)	5 (41.7)	9 (14.5)	0.010
Types of bleeding *	Melena	0 (0.0)	2 (16.7)	2 (3.2)	0.035
	Hematuria	2 (4.0)	0 (0.0)	2 (3.2)	1.000
	Others	1 (2.0)	0 (0.0)	1 (1.6)	1.000
Lymph node enlargement *		0 (0.0)	1 (8.3)	1 (1.6)	0.194
Leukopenia *		47 (94.0)	9 (75.0)	56 (90.3)	0.081
Thrombocytopenia *		48 (96.0)	11 (91.7)	59 (95.2)	0.482

Values are presented as numbers (%). * Fisher’s exact test. ^†^ Mann–Whitney *U* Test. IQR, interquartile range.

## Data Availability

Not applicable.
